# *In vivo* measured joint friction in hip implants during walking after a short rest

**DOI:** 10.1371/journal.pone.0174788

**Published:** 2017-03-28

**Authors:** Philipp Damm, Alwina Bender, Georg Duda, Georg Bergmann

**Affiliations:** Julius Wolff Institute, Charité–Universitätsmedizin Berlin, Berlin, Germany; University of Zaragoza, SPAIN

## Abstract

**Introduction:**

It has been suspected that friction in hip implants is higher when walking is initiated after a resting period than during continuous movement. It cannot be excluded that such increased initial moments endanger the cup fixation in the acetabulum, overstress the taper connections in the implant or increase wear. To assess these risks, the contact forces, friction moments and friction coefficients in the joint were measured *in vivo* in ten subjects. Instrumented hip joint implants with telemetric data transmission were used to access the contact loads between the cup and head during the first steps of walking after a short rest.

**Results:**

The analysis demonstrated that the contact force is not increased during the first step. The friction moment in the joint, however, is much higher during the first step than during continuous walking. The moment increases throughout the gait cycle were 32% to 143% on *average* and up to 621% *individually*. The high initial moments will probably not increase wear by much in the joint. However, comparisons with literature data on the fixation resistance of the cup against moments made clear that the stability can be endangered. This risk is highest during the first postoperative months for cementless cups with insufficient under-reaming. The high moments after a break can also put taper connections between the head and neck and neck and shaft at a higher risk.

**Discussion:**

During *continuous* walking, the friction moments *individually* were extremely varied by factors of 4 to 10. Much of this difference is presumably caused by the varying lubrication properties of the synovia. These large moment variations can possibly lead to friction-induced temperature increases during walking, which are higher than the 43.1°C which have previously been observed in a group of only five subjects.

## Introduction

Total hip joint replacement is performed more than 200,000 times in Germany alone [1]. During recent decades, the patients became younger and more active. Hence, their demands for functionality and lifetime of the implants have increased. Loosening of the artificial cup and inlay is one of the most common reasons for the failure of total hip replacements [2–4]. Polyethylene wear and aseptic loosening of the cup account for 26% and 48%, respectively, of reoperations [2,5]. Another study demonstrated that 30% to 40% of all revisions require a change of cup or inlay [6].

Wear is caused by friction, and aseptic loosening can be due to moments that stress the fixation in the acetabulum. These moments are not only determined by the patient’s activities and, thus, the frequency and magnitude of the contact forces but also by the amount of friction in the joint. *In vitro* studies using different test conditions [7–18] demonstrated that the materials of the implant head and inlay primarily influenced the friction. In several studies, the stability of cup-bone bonding was investigated in cadavers or using plastic bone substitutes. A loosening moment of 8.8 Nm was reported for cementless cups [19], but values as low as 2.2 Nm were reported [20], both with an under-reaming of 1 mm. In comparison, our own i*n vivo* load measurements with instrumented hip implants [21] have determined average friction moments during walking between 2.25 ±0.29 Nm (three months postoperatively) and 1.76 ±0.83 Nm (12 months postoperatively) [22,23]. This indicates that already during typical activities of daily living, such as walking, critical friction moments can occur in total hip joint replacements.

Analogous to higher moments in technical joints after movement started, it was suspected that friction in joint implants may also be higher after a short break, during which time the lubrication film breaks down [24,25]. Based on *in vivo* measurements [26] of typical activity times and resting periods in hip patients and using a pin-on-ball test, joint friction was investigated *in vitro* after movement began [24]. Friction after 5 s resting was 30% higher in ceramic-UHMWPE pairings than during the following continuous movement. The increase depended on the tribological pairing of the implant and correlated to the rest time. The results of this study confirmed that the peak moments in hip implants are higher after a rest than during continuous movements and may jeopardize the cup fixation. However, the *in vitro* data cannot directly be applied to *in vivo* situations because the kind of movement, the force amplitudes and the lubricant are different. The lubricating property of the synovia has an especially large influence on friction in the joint, as a difference of up to 451% in the friction moments in a group of ten subjects suggests [23]. Such individual differences of lubrication may also effect the possibly increased moments after a rest.

The main goal of this study was to obtain *in vivo* data on the increases of friction moments and friction coefficients after joint movement began. This enables the estimation of the potential risk of cup loosening due to high moments. For this purpose, contact forces and friction moments in instrumented hip implants of ten subjects were measured during walking, and the friction coefficients were calculated from these data.

## Methods

### Instrumented hip implant

To measure friction between the head and cup *in vivo*, instrumented hip implants were used [21]. The titanium implant (CTW, Merete Medical, Berlin; Germany) was combined with a 32 mm Al_2_O_3_ ceramic head and an XPE inlay. The neck of this clinically proven standard implant was modified to house an inductive power supply, six strain gauges, signal amplifiers and telemetric data transmission [21]. The external measurement system [27,28] supplied the inductive coil around the patient’s hip joint. The received signals were recorded simultaneously with the patient’s images on video tape.

#### Joint contact forces and friction moments

The femur-based coordinate system [29] was located in the head center of a right-sided implant [30]. Data from left implants were mirrored to the right side. The **resultant joint contact force F**_**res**_ was calculated from the three force components in the lateral (F_x_), anterior (F_y_), and superior (F_z_) directions. The **resultant friction moment M**_**res**_ was determined from the three components M_x_, M_y_, M_z_, rotating positively around the corresponding axes.

### Calculation of coefficient of friction

Based on all force and moment components, the magnitude of the three-dimensional **coefficient of friction μ** was calculated [23], assuming Coulomb friction and a head radius R:

$$\mu ={\text{M}}_{\text{res}}/\left({\text{H }*\text{ F}}_{\text{res}}\right)$$ (1)

The lever arm H is given by the following equation, see also [23]

$$\underline{\text{H}}=\text{R }*\;[{\underline{\text{F}}}_{res}/F_{res}-\text{ cos }(\underline{\text{R}},{\underline{\text{M}}}_{res})\;*\;({\underline{\text{M}}}_{res}/M_{res})]$$ (2)

The coefficient μ was only determined for F_res_ ≥ 25%BW and M_res_ ≥ 0.02%BWm to ascertain an accuracy of μ better than 5%.

### Patients and measurements

Ten patients with instrumented implants participated in the study (Table 1). They gave their informed written consent to participate. The study was approved by the ethical committee of the Charité–Universitätsmedizin Berlin, Germany (EA2/057/09) and was registered in the German Clinical Trials Register (DRKS00000563). The measurements were performed an average of 17 months (12–31 months) postoperatively during 10m of level walking at a self-selected walking speed. Five to eighteen trials per subject were recorded. The subjects stood still on both legs for 12 s, on average, before they started walking with the ipsilateral leg.

**Table 1:** Investigated subjects and the measurement parameters.

Subject	Bodyweight	Measurement	Ø Rest	Trials	Gender	Age
	[N]	[months post OP]	[s]			[years]
H1L	760	13	5	6	male	56
H2R	767	12	7	6	male	62
H3L	1.096	12	11	18	male	60
H4L	796	12	7	11	male	51
H5L	863	31	14	10	female	65
H6R	856	24	18	8	male	70
H7R	899	24	21	5	male	54
H8L	874	18	20	10	male	57
H9L	1197	13	5	11	male	55
H10R	995	12	10	9	female	54
**Average**	**910**	**17**	**12**	**10**	**-**	**58**

### Data evaluation

**Fig 1:**
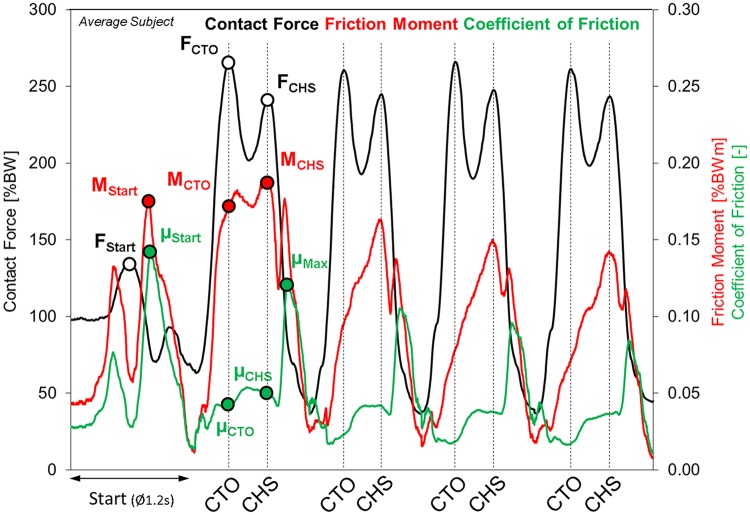
Resultant force F_res_, resultant friction moment M_res_ and friction coefficient μ before “Start” and during four steps after rest. Data from average subject.

**Fig 2:**
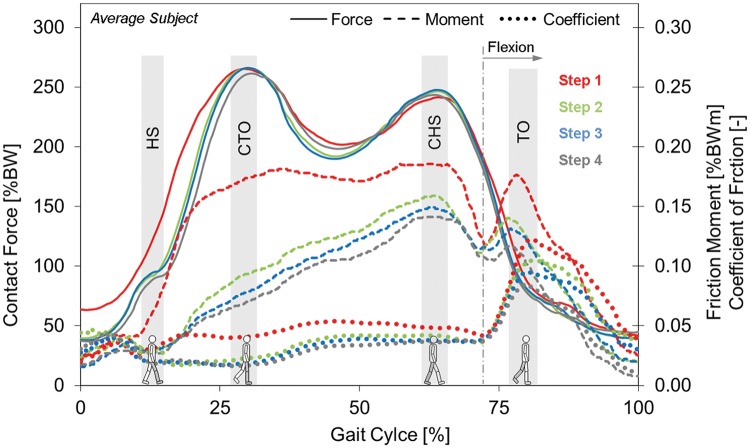
Resultant joint contact force F_res_, resultant friction moment M_res_ and coefficient of friction μ for steps 1 to 4 after rest. M_res_ and μ are higher during step 1 (red lines) than during the following steps. **F**_**res**_ is higher only until CTO. HS = ipsilateral heel strike, CTO = contralateral toe off, CHS = contralateral heel strike, TO = ipsilateral toe off. Data from average subject after average rest time of 12 s.

All forces were determined as a percent of the patient’s bodyweight (**%BW**); the friction moments in %BW*m. For a subject with a body weight of 100 kg, as an example, the values must be multiplied by a factor of 9.81 to obtain numbers in N or Nm. The continuous time patterns and the numerical peak values of forces and moments were analyzed separately for each of the first four steps after rest. Each complete step started and ended at the instants when F_res_ became a minimum (Figs 1 and 2). The ‘Start’ phase, preceding the first step, started when the ipsilateral leg started to move after stance and ended when F_res_ became a minimum before heel strike. If not mentioned as being ‘*individual’*, all reported data refer to results from the *average* subject.

*Time patterns*: First, the durations of the Start phases and the four steps were averaged (Fig 1) from all individual trials (Table 1). Then, the time patterns of all six force and moment components and the results from all trials were averaged, separately for the Start phase and the four steps. For averaging, a dynamic time warping procedure was applied [31], which delivered an output that retains the typical maxima and minima of the included signals. Finally, the obtained *individual* averages were averaged again from all subjects to obtain the load-time behavior of an *average* subject (Fig 2). The friction coefficient μ throughout each trial was calculated from these *individual* or *average* force and moment components.

*Numerical values*: For the Start phase (Fig 1), the absolute maxima **F**_**start**_, **M**_**start**_, **and μ**_**start**_ of the resultant force, the resultant moment and the friction coefficient were analyzed. During each of the following steps, the curves of the force F_res_ always exhibited two peak values **F**_**CTO**_ and **F**_**CHS**_ (Figs 1 and 2), at approximately the instant of contralateral toe off (CTO) and contralateral heel strike (CHS). One of both peak values was always the absolute maximum of F_res_. For the moment M_res_, the two values **M**_**CTO**_ and **M**_**CHS**_ were determined at the same instants as the peak forces. The maximum resultant moment **M**_**max**_ throughout the entire cycle duration mostly acted *very shortly* after M_CHS_ and is about 10% to 15% higher (Table 2). Two numerical values **μ**_**CTO**_ and **μ**_**CHS**_ of the friction coefficient were also identified at the instants of the two peak forces. The absolute maximum **μ**_**max**_ of the friction coefficient was denoted as μ_max_.

**Table 2:** Resultant forces and moments plus coefficient of friction during the first four steps after rest. Data from individual and average subjects at the instants of contralateral toe off (CTO) and contralateral heel strike (CHS) plus maximum values throughout the entire gait cycle. “Start” = maximum values before complete step 1 started. SD = standard deviation. Minima and maxima indicated in bold.

Subject	F_Start_	M_Start_	μ_Start_	F_CTO_	F_CHS_	M_CTO_	M_CHS_	M_MAX_	μ_CTO_	μ_CHS_	μ_max_	F_CTO_	F_CHS_	M_CTO_	M_CHS_	M_MAX_	μ_CTO_	μ_CHS_	μ_max_
	[%BW]	[%BWm]	[1]	[%BW]	[%BW]	[%BWm]	[%BWm]	[%BWm]	[1]	[1]	[1]	[%BW]	[%BW]	[%BWm]	[%BWm]	[%BWm]	[1]	[1]	[1]
	**Start**			**Step 1**											**Step 2**				
**H1L**	**92**	0.195	**0.268**	213	**186**	0.258	0.241	0.263	0.076	0.083	0.204	207	**188**	0.161	0.214	0.222	0.049	0.072	0.187
**H2R**	121	0.229	0.221	**204**	222	0.137	0.177	0.198	0.042	0.051	0.224	**206**	231	**0.029**	0.141	0.180	**0.009**	0.039	0.233
**H3L**	128	0.178	0.170	223	233	0.202	0.206	0.299	0.057	0.057	0.189	220	239	0.096	0.158	0.225	0.028	0.042	0.238
**H4L**	93	**0.091**	**0.117**	224	211	**0.097**	0.168	0.179	0.028	0.051	0.115	228	220	0.057	0.110	**0.111**	0.016	0.032	0.129
**H5L**	155	0.262	0.130	**389**	276	0.258	0.328	0.353	0.043	0.048	0.263	**400**	282	0.118	0.238	0.286	0.028	0.050	**0.382**
**H6R**	134	0.208	0.177	252	255	0.122	**0.118**	0.145	0.026	0.036	0.118	252	260	0.097	**0.105**	0.141	0.021	0.030	0.144
**H7R**	**223**	**0.425**	0.224	349	**289**	0.107	0.140	0.181	0.042	0.075	0.175	359	**288**	0.082	0.107	0.151	0.019	0.054	0.160
**H8L**	129	0.216	0.138	319	288	0.208	0.211	0.217	0.031	**0.029**	**0.101**	308	282	0.133	0.220	0.229	0.025	0.026	**0.090**
**H9L**	129	0.154	0.150	243	213	0.105	0.119	**0.136**	**0.021**	0.031	0.127	259	228	0.085	0.107	0.113	0.016	**0.024**	0.096
**H10R**	166	0.240	0.168	242	235	**0.303**	**0.380**	**0.387**	**0.080**	**0.105**	**0.271**	230	238	**0.264**	**0.416**	**0.428**	**0.073**	**0.115**	0.317
**Min**	92	0.091	0.117	204	186	0.097	0.118	0.136	0.021	0.029	0.101	206	188	0.029	0.105	0.111	0.009	0.024	0.090
**Max**	223	0.425	0.268	389	289	0.303	0.380	0.387	0.080	0.105	0.271	400	288	0.264	0.416	0.428	0.073	0.115	0.382
***Average***	***137***	***0*.*220***	***0*.*176***	***266***	***241***	***0*.*180***	***0*.*209***	***0*.*236***	***0*.*045***	***0*.*057***	***0*.*179***	***267***	***246***	***0*.*112***	***0*.*182***	***0*.*209***	***0*.*029***	***0*.*048***	***0*.*197***
***SD***	38	0.087	0.048	60	33	0.072	0.083	0.082	0.019	0.023	0.059	64	30	0.062	0.092	0.091	0.018	0.026	0.091
				**Step 3**								**Step 4**							
**H1L**				**204**	**198**	0.130	0.206	0.217	0.041	0.066	0.181	209	**194**	0.115	0.202	0.219	0.035	0.066	0.172
**H2R**				210	231	**0.023**	0.149	0.181	**0.007**	0.041	0.224	**202**	232	**0.019**	0.149	0.189	**0.007**	0.041	0.230
**H3L**				219	241	0.103	0.168	0.232	0.030	0.044	0.216	219	240	0.102	0.171	0.231	0.029	0.045	0.215
**H4L**				232	215	0.054	0.105	0.108	0.015	0.031	0.148	232	218	0.055	0.102	0.103	0.015	0.030	0.213
**H5L**				**400**	**293**	0.110	0.188	0.245	0.022	0.049	0.245	**358**	271	0.073	0.148	0.174	0.021	0.049	0.195
**H6R**				248	263	0.079	0.096	0.132	0.020	0.027	0.118	251	249	0.074	**0.084**	0.115	0.021	0.030	0.104
**H7R**				350	291	0.085	**0.094**	0.145	0.019	0.040	0.146	357	**289**	0.088	0.092	0.142	0.016	0.036	0.129
**H8L**				313	277	0.103	0.215	0.216	0.022	0.023	**0.088**	310	285	0.094	0.221	0.225	0.020	0.022	0.091
**H9L**				250	221	0.078	0.096	**0.101**	0.017	**0.021**	0.093	241	208	0.080	0.095	**0.096**	0.017	**0.021**	**0.083**
**H10R**				238	234	**0.258**	**0.399**	**0.433**	**0.070**	**0.110**	**0.314**	240	238	**0.257**	**0.398**	**0.418**	**0.069**	**0.111**	**0.361**
**Min**				204	198	0.023	0.094	0.101	0.007	0.021	0.088	202	194	0.019	0.084	0.096	0.007	0.021	0.083
**Max**				400	293	0.258	0.399	0.433	0.070	0.110	0.314	358	289	0.257	0.398	0.418	0.069	0.111	0.361
***Average***				***266***	***246***	***0*.*102***	***0*.*172***	***0*.*201***	***0*.*026***	***0*.*045***	***0*.*177***	***262***	***242***	***0*.*096***	***0*.*166***	***0*.*191***	***0*.*025***	***0*.*045***	***0*.*179***
***SD***				62	31	0.059	0.088	0.092	0.017	0.025	0.069	55	30	0.059	0.090	0.090	0.016	0.025	0.080

Separately for the Start phase and the four steps, these distinct force, moment and coefficient values were first determined numerically from the time patterns of the single trials and then averaged in the same sequence as the load-time patterns. First, the *individual* averages were calculated and then, based on the obtained numbers, the values for the *average* subject plus the corresponding minima, maxima and standard deviations. Information about the variations of all parameters in the individuals and their average is supplied by the reported standard deviations. The procedure used for averaging the *time patterns* minimized the summed errors between all included patterns throughout the entire measurement time. Therefore, the peak values in the *curves* of the average subject (Fig 2) can slightly deviate from the corresponding, *numerically* averaged peak values (Table 2).

To determine whether the values of the eight F_res_, M_res_ and μ measurements during the first step were different from the corresponding values during the last step, each measurement from step 1 of the individual and average subjects was compared to the value from step 4. The obtained differences in percent were statistically analyzed (Wilcoxon, p ≤ 0.05).

*Rest times*: To investigate whether the duration of the rest time influenced the changes of M_res_ and μ, the individually averaged rest times per trial (Table 1) were correlated to the changes of the corresponding six M_res_ and μ values between steps 1 and 4.

## Results

### Joint contact forces F_res_

Time patterns: During the early gait phase until CTO, F_res_ was much higher during step 1 than during steps 2 to 4 (Fig 2). This surplus was 59% when the steps started and 35% at ipsilateral heel strike (HS). After reaching CTO and until the end of the cycles, F_res_ was nearly the same for all four steps.

**Fig 3:**
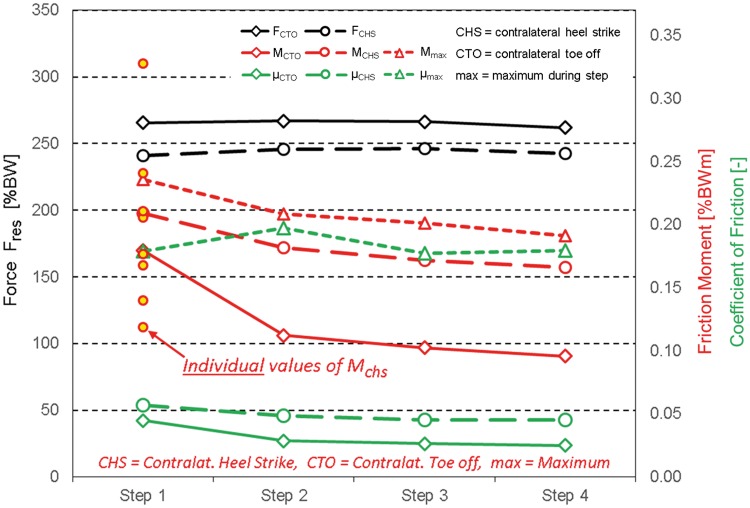
Contact forces F_res_, friction moments M_res_ and coefficients of friction μ during first four steps after rest. Values at the instant of contralateral toe off (CTO) and contralateral heel strike (CHS) plus maxima (max) during the entire step time. Averages from ten subjects. Small circles = illustration of large individual variation of M_CHS_ during step 1; other variations see Table 2.

Numerical values: The *individual* variations of the two force peaks F_CTO_|F_CHS_ were large (Table 2). For each of the four steps the standard deviations were approximately 23|13% of the average values. *From step to step*, both *average* force peaks were nearly unchanged (Table 2, Figs 2 and 3). F_CTO_ during step 1 exceeded the value from step 4 by only 1.3% (Table 3); for F_CHS_ this difference was -0.8%, that is, F_CHS_ was slightly smaller during step 4 than step 1.

**Table 3:** Increases of forces, moments and coefficient of friction during the first step after walking. Increase in percent of values during step 1 relative to values during step 4. Minima und maxima indicated in bold, p-values: Wilcoxon test.

Subject	F_CTO_	F_CHS_	M_CTO_	M_CHS_	M_MAX_	μ_CTO_	μ_CHS_	μ_max_
	[%]	[%]	[%]	[%]	[%]	[%]	[%]	[%]
H1L	1.9	-4.1	124	19	20	116	26	19
H2R	1.0	**-4.3**	**621**	19	5	**542**	23	-3
H3L	1.8	-2.9	98	20	29	94	28	-12
H4L	**-3.4**	-3.2	76	65	74	84	73	**-46**
H5L	**8.7**	1.8	253	**122**	**103**	104	-2	35
H6R	0.4	**2.4**	65	40	26	23	21	14
H7R	-2.2	0.0	22	52	27	160	**105**	36
H8L	2.9	1.1	121	**-5**	-4	57	34	11
H9L	0.8	2.4	31	25	42	25	52	**54**
H10R	0.8	-1.3	**18**	**-5**	**-7**	**16**	**-5**	-25
*Min*	-3.4	-4.3	18	-5	-7	16	-5	-46
*Max*	8.7	2.4	621	122	103	542	105	54
***Average***	***1*.*3***	***-0*.*8***	***143*.*0***	***35*.*4***	***31*.*5***	***122*.*1***	***35*.*5***	***8*.*3***
*SD*	3.1	2.6	172	36	33	147	32	29
p-value	0.201	0.285	0.005	0.013	0.22	0.005	0.014	0.721

### Friction moments M_res_

Time patterns: The friction moment M_res_ had a different time behavior than F_res_ and revealed only one maximum at or shortly after CHS (Fig 1). During step 1, M_res_ rose sharply after heel strike until CTO, while F_res_ increased, and then changed only a little until CHS (Fig 2). In contrast to this, M_res_ increased nearly linearly between HS and CHS during the following steps. After CHS, M_res_ always fell, approximately until the joint movement changed from extension to flexion. This instant was controlled by the synchronous videos. Then, it rose to an intermediate peak value, most pronounced for step 1, and continuously fell until the step ended. During the entire cycle time, M_res_ was higher during step 1 than later.

Numerical values: In all *subjects*, except H10R, all three moment values, M_CTO_, M_CHS_ and M_max_ were higher during step 1 than later (Table 2). The *individual* variations of all three moments, were large. For step 1, for example, the standard deviations were 39%, 38% and 33% of the average values. The small red circles shown in Fig 3 illustrate the huge range of *individual* values of M_CHS_ during step 1. During the next three steps the standard deviations were even higher. In the *average* subject, however, uniform step to step changes were observed (Table 2). M_CTO_ fell from step to step (Fig 3) and was most pronounced from step 1 to 2. With 143% (p = 0.005), the surplus from step 1 relative to step 4 was very large (Table 3). M_CHS_ was much higher than M_CTO_ and decreased continuously but was less pronounced than M_CTO_ from step 1 to 2. The value during step 1 was only 35% (p = 0.013) higher than during step 4. The maximum moment M_max_ only slightly exceeded M_CHS_. The step to step changes of M_max_ were similar to those of M_CHS_, with a total surplus during step 1 of 32% relative to step 4. The moment courses in Fig 3 indicate that all three friction moments will probably only slightly decrease further after step 4.

### Coefficient of friction μ

Time patterns: The charts of μ from the *average* subject (Figs 1 and 2) show that it was permanently higher during step 1 than during the following steps. During step 1, μ in the *average* subject already rose at HS and stayed at a high level after CTO. The rise during the following three steps only started after CTO. When hip flexion began, μ was nearly the same for all four steps. It then uniformly and sharply increased to the absolute maxima at around ipsilateral toe off (TO), which were more than twice as high than the values during the whole stance phases. After TO, μ continuously decreased during the remaining swing phase.

Numerical values: All three *individual* friction values, μ_CTO_, μ_CHS_ and μ_max_, varied a lot, as observed from the ranges and standard deviations in Table 2. In subject H10R, all three friction values were much higher than in all other patients. The *individual* values of μ_CTO_|μ_CHS_ exceeded the *average* ones by up to 176%|239%

In the *average* subject, μ_CTO_ was 122% (p = 0.005) higher during the first than during the last step (Table 3, Fig 3). For μ_CHS_, this surplus was 36% (p = 0.014). The extreme *individual* surplus of μ_CHS_ from step 1 to 4 was 105%, observed in subject H7R. The maximum coefficient μ_max_ was on average by only 8% higher during step 1 than step 4. The declines in all three μ values with the step number (Fig 3) was less pronounced than the drops observed for the moments. During step 2, μ_max_ was even larger than during step 1, an effect observed for six of the ten *individual* subjects.

### Start phase

During the Start phase, the patterns of F_res_, M_res_ and μ were different from those during step 1 (Fig 1). The numerical value of F_start_ (Table 2) was on average 48% lower, compared to F_CTO_ during step 1, with individual variations between -60% to -32%. The values M_start_|μ_start_, however, were on average 3%|7% higher, compared to F_CTO_ during step 1. However, these changes again varied a lot, with ranges of -49% to +135% for M_start_ and -51% to +50% for μ_start_.

### Rest times

No or very poor correlations existed between the individually averaged rest times (Table 1) and the six values of M_res_ and μ (Table 3); R^2^ was always below 0.23.

## Discussion

High friction moments in hip implants increase wear in the joint, especially during the early postoperative weeks, when the fixation stability of cementless implants is lower than later; high friction moments can possibly also endanger the fixation of the cup. It was shown [30] that the peak friction moment during some activities can already reach values that were reported in the literature to jeopardize the cup fixation. [24,25] It has been reported that the *in vitro* friction moments are higher during the first loading cycle after a rest than during continuous movement. This indicated that frequently increased moments after rests might increase the risk for cup loosening or lead to more wear. Because the test conditions in these studies were not realistic, we examined whether the friction moments and the coefficient of friction are higher *in vivo* when walking starts after a rest.

With regard to the reported large individual variations of all load parameters, the average values cannot be generalized. The friction coefficient μ_CTO_, for example, was by 542% larger during step 1 than step 4 in one subject, but by only 16% in another one. The low significance of the load changes is indicated by the low p-values in Table 2. However, decreases of all moments and friction coefficients except μ_max_ were observed in at least eight of ten subjects. Therefore, the moment and friction increases after a rest prior to walking can be expected for the majority of subjects, but their extend cannot be predicted exactly for a specific individuum. Other limitations to this study are the small number of ten investigated subjects and that only one tribological paring was investigated (Al_2_O_3_/XPE).

### Friction moment and coefficient

During the first step after standing, the friction moment M_res_ at the instant of the first|second force maximum was 143%|35% higher than during step 4. At the same time, the friction coefficient μ from step 1 exceeded that from step 4 by 122%|36%. This means that the decreases of M_res_ and μ throughout the first four steps are approximately proportional.

Figs 1 and 2 show that M_res_ and μ, during the initial step, rise sharply after heel strike, when the contact force F_res_ increases, and stay at increased levels until the CHS, when F_res_ falls again. This behavior is in sharp contrast to the changes of M_res_ and μ during continuous walking (assumed to be represented by step 4), when both measures increase continuously throughout the whole stance phase. The force F_res_ during step 1 is only initially higher than later.

Possible explanations for these observations are as follows: During the initial rest, all or most of the synovia is squeezed out of the joint, leading to a nearly non-lubricated contact between head and cup surfaces. Throughout the first step, until toe off, no synovia can be transported back into the contact zone because this area is pressurized by the high contact force F_res_. When F_res_ falls after toe off, the joint movement during the swing phase transports synovia back into the joint. The second and all following steps, therefore, start with a sufficient lubricating film. The high contact force then again reduces the lubricating film throughout the stance phase, which leads to the continuous increase of M_res_ and μ. The high values of F_res_ in the beginning of step 1 are probably required for accelerating the body to walking speed.

If these explanations are valid, the friction moments during the one legged stance, when small joint movements cannot be avoided, should also be high. This assumption is confirmed by some exemplary measurements in the public data base OrthoLoad.com (parameters: Implant = ‘Hip Joint III’ and Activity = ‘One Legged Stance’). Such high moments will be the focus of a future study.

With 0.045|0.057, the *average* values of μ_CTO_|μ_CHS_ during step 1 were very close to the maximum of 0.055 found in simulator tests [25]. Only μ_max_, acting at TO when F_res_ had already fallen to approximately one-third of the two maxima, was three times higher than this literature value. Because all three μ values have fallen to nearly constant levels until step 4; the numbers from this step can be compared to those previously reported for continuous walking [22,23].

All subjects received implants with the same tribological pairing (Al_2_O_3_/XPE). Nevertheless, the friction moments and the coefficients of friction *individually* varied a lot. Data from step 4, assumed to be representative for continuous walking, demonstrated variations of M_CTO_|M_CHS_|M_max_ by factors of 10|5|4. For μ_CTO_|μ_CHS_|μ_max_, these factors were nearly identical. Subject H10R especially stands out as the moment and coefficient values were always extremely higher than those in the other subjects. The three values of M_res_ and μ for H10R exceeded the averages from all subjects by up to 167%|239%. Such large variations are probably caused by different individual lubrication conditions, which can be influenced by (i) the lubricating quality of the synovia [14], (ii) the roughness of the gliding surfaces [14,32], (iii) the joint clearance [14,32,33] and (iv) the orientation of the acetabular cup [14,34], which influences the load transmitting area. Data on the impact of other factors, such as the sliding speed or joint contact area, will be investigated in a further study.

### Wear

Frequent reasons for revisions of hip joint replacements are still wear and pathological reactions to wear particles [2,5,35]. Simulator studies demonstrated a correlation between the friction between sliding partners and the wear rates [33]. In theory, increased friction moments and coefficients during the first step of walking could, therefore, increase the wear. However, during walking and other repetitive activities, a single starting cycle with high moments is typically followed by many continuous loading cycles with lower moments. Because the wear volume not only depends on the height of the friction moment but also on the number of loading cycles, much increased wear due to increased moments after rest should not be expected.

### Cup loosening

The primary and long-term stability of the cup fixation depends, except for the height of the friction moments, on the quality of the cup-bone interface, the fixation technique, the type of porous coating and the bone quality. Simulator studies demonstrated that insufficient under-reaming of the acetabulum decreases the primary fixation stability (Curtis M. J. et al., 1992; Tabata et al., 2015). With an under-reaming of only 1 mm, the loosening moments lay between 2.2 and 8.8 Nm. The *average* maximum moment of M_res_ = 2.15Nm (0.236%BWm, assumed BW = 1000 N), reported here for step 1 just meets the lowest reported value. However, the highest *individual* moment from subject H10L was 4.28 Nm (0.43%BWm, BW = 995 N) and this is much higher than the lowest stability level reported in the literature.

An additional risk factor, not considered in the current study, is the fact that joint friction changes during the first months after replacement (Damm et al. 2015). Three months after surgery, M_res_ was on average by 47% higher than after 12 months. The extreme moment value in H10L would then have further risen to 6.33 Nm. Possible factors for higher postoperative friction may be a still lacking smoothening of the gliding surfaces and initially insufficient synovia properties. Therefore, even higher values than reported here must be expected shortly after surgery.

The high maximum *in vivo* friction moments and the lowest reported *in vitro* loosening moments together indicate that the higher moments at the beginning of walking can put the cup fixation at risk in subjects with high body weight, inferior lubricating properties of the synovia and cementless cups, which are inserted with too small under-reaming. This risk is highest during the first postoperative months.

### Joint temperature

High temperatures in artificial joints could be a potential risk factor for the longevity of the implant system. For the combination of conventional polyethylene and ceramic cups with ceramic and metal heads, friction-induced temperature increases up to 43.1°C after one hour of walking were reported [36]. These increases varied dramatically and individually, depending on the lubrication properties of the synovia [37] and the tribological materials.

By the same reasons as for the wear a distinct influence of increased moments after breaks on the implant temperature during walking can be excluded. However, the current study again demonstrates that friction moments and coefficients are extremely varied from person to person. The three moment measures individually differed by factors of 4 to 10. A factor of four approximately corresponds to the individual differences of temperature increases measured *in vivo* during walking [36]. The higher factor of ten let us assume that the implant temperature may rise much more in some subjects than observed previously.

### Head-stem connection

Friction influences the mechanical stress in the head-neck taper region and, if existent, the neck-stem connection. Up to 143% higher friction moments after a rest, compared to continuous walking, will lead to an increase of these stresses. Therefore, higher friction after a rest can be a potential risk factor for the mechanical stability of suboptimal taper connections. If the mechanical connection becomes loose, micromotion between the components increases and this begins a corrosion cascade [38,39] that can eventually cause implant loosening. Furthermore, increased wear and corrosion products provoke various biological and chemical effects in the surrounding tissues [39,40], which also lead to implant failure.

## Conclusion

It was demonstrated that hip joint friction during the first step of walking after a rest is much higher than that during continuous movement. The initial friction moments were raised on *average* by 35% to 143% and *individually* by up to 621% compared to continuous walking.

### Wear and cup loosening

The higher moments will probably increase wear in the joint only very slightly, but they can endanger the fixation of the cup in the acetabulum. This risk is highest for cementless cups with insufficient under-reaming and during the first postoperative months.

### Head-stem connection

The high moments when walking begins can also put taper connections between the head and neck and between the neck and shaft at a higher risk.

### Joint temperature

In five subjects, friction-induced temperature increases up to 43.1°C were observed in hip implants during continuous walking [36]. There was much individual variation, and this was explained by different lubricating properties of the synovia. The measured friction moments during continuous walking, reported here, *individually* varied by factors up to 10, which is more than the differences of the reported temperature increases. Therefore, it seems worthwhile to perform another investigation with a larger group of patients to determine whether even higher implant temperatures during walking may endanger the long-term outcome of the replacement.

## Additional data

Selected examples of the *in vivo* measurements, on which this study was based, are published in the public data base www.orthoload.com.
